# Exploiting B Cell Transfer for Cancer Therapy: Engineered B Cells to Eradicate Tumors

**DOI:** 10.3390/ijms22189991

**Published:** 2021-09-16

**Authors:** Audrey Page, Julie Hubert, Floriane Fusil, François-Loïc Cosset

**Affiliations:** CIRI-Centre International de Recherche en Infectiologie, Univ Lyon, Université Claude Bernard Lyon 1, Inserm, U1111, CNRS, UMR5308, ENS Lyon, 46 Allée d’Italie, F-69007 Lyon, France; audrey.page1@ens-lyon.fr (A.P.); julie.hubert@ens-lyon.fr (J.H.); floriane.fusil@ens-lyon.fr (F.F.)

**Keywords:** adoptive cell transfer, B cells, immunotherapy, gene editing, antigen presentation, antibody

## Abstract

Nowadays, cancers still represent a significant health burden, accounting for around 10 million deaths per year, due to ageing populations and inefficient treatments for some refractory cancers. Immunotherapy strategies that modulate the patient’s immune system have emerged as good treatment options. Among them, the adoptive transfer of B cells selected *ex vivo* showed promising results, with a reduction in tumor growth in several cancer mouse models, often associated with antitumoral immune responses. Aside from the benefits of their intrinsic properties, including antigen presentation, antibody secretion, homing and long-term persistence, B cells can be modified prior to reinfusion to increase their therapeutic role. For instance, B cells have been modified mainly to boost their immuno-stimulatory activation potential by forcing the expression of costimulatory ligands using defined culture conditions or gene insertion. Moreover, tumor-specific antigen presentation by infused B cells has been increased by ex vivo antigen loading (peptides, RNA, DNA, virus) or by the sorting/ engineering of B cells with a B cell receptor specific to tumor antigens. Editing of the BCR also rewires B cell specificity toward tumor antigens, and may trigger, upon antigen recognition, the secretion of antitumor antibodies by differentiated plasma cells that can then be recognized by other immune components or cells involved in tumor clearance by antibody-dependent cell cytotoxicity or complement-dependent cytotoxicity for example. With the expansion of gene editing methodologies, new strategies to reprogram immune cells with whole synthetic circuits are being explored: modified B cells can sense disease-specific biomarkers and, in response, trigger the expression of therapeutic molecules, such as molecules that counteract the tumoral immunosuppressive microenvironment. Such strategies remain in their infancy for implementation in B cells, but are likely to expand in the coming years.

## 1. Introduction

Cancers are currently considered a global health priority, causing around 10 million deaths per year, thus representing the second most common cause of death (15%). The therapeutic approaches that are available rely mainly on chemotherapy, radiotherapy and surgery. Despite huge progress in the previous decades, these treatments do not cure or prevent disease flares for many cancers. Thus, there is an urgent need for new safer, less invasive and more efficient therapies. In this context, immunotherapies, and more particularly adoptive cell transfer, have emerged as promising solutions for eradicating tumor cells, by exploiting the patient’s immune system and enhancing its abilities to fight cancer cells. Such therapies are believed to be less toxic systemically and might also reduce auto-immunity issues. The ultimate goal of adoptive cell therapies for cancer is to trigger the development of antitumor adaptive responses in the patient. To mount such immune responses, the injected cells must be able to activate their appropriate effector cell counterparts (mainly T cells), leading to an efficient accumulation of immune effector cells at the tumor site, in order to overcome the immunosuppressive tumor microenvironment. The infused cells can be collected from the patient directly (autologous cells) or can originate from a healthy donor subject (allogenic cells), who needs to be human leucocyte antigen (HLA)-compatible with the patient to avoid graft rejection. In the future, induced pluripotent stem cells could also be used to overcome this allo-incompatibility problem.

Several cell types may be suitable for such therapeutic approaches. Among them, T cells have been the most widely studied. Of note, the engineering of CAR (Chimeric Antigen Receptor) T cells to trigger tumor recognition and killing, as well as the activation of other antitumor immune cells showed great promise for the treatment of blood cancers. However, although T cells present numerous advantages for adoptive cell therapies in terms of toxicity and ex vivo proliferation capacity, B cells should not be underestimated, because several of their intrinsic properties also make them excellent candidates for cancer treatments and cures. Indeed, B cells are professional antigen-presenting cells (APC) that can present antigens not only on major histocompatibility complex I (MHC-I) molecules, but also on major complex histocompatibility II (MHC-II) molecules after antigen recognition on the B cell receptor (BCR) and endocytosis of the BCR-antigen complex. Such presentations combined to the expression of costimulatory molecules can activate T cells that are specific to the presented antigen, after the homing of B cells to secondary lymphoid organs. Activated T cells then destroy tumor cells either directly by the release of lytic enzymes (mainly CD8+ cytotoxic T cells), or indirectly by interaction with other immune cells.

Naïve and memory B cells express a membrane-anchored BCR specific to a given antigen. After recognition of the antigen by the BCR and B cell-activation and differentiation into plasma cells, immunoglobulins are secreted (antibodies). These antibodies specific to tumor antigens, for instance, can recognize cancer cells through their variable regions and mediate tumor killing by phagocytosis (antibody-dependent cell phagocytosis: ADCP), by lysis induced by CD8+ T cells or NK cells (antibody-dependent cell cytotoxicity: ADCC) or by the complement (complement-dependent cytotoxicity: CDC) through their constant regions recognized by Fc receptors expressed on immune cells. Moreover, plasma cells have a high capacity to secrete proteins, which might be very useful for adoptive cell transfer. In parallel, upon encountering their specific antigen, B cells can differentiate and an immunological memory is therefore established, generating a long-term protective immunity. Of high importance, B cells from cancer patients retain the ability to proliferate, present tumor antigens and recognize homing cytokines in a range comparable to the B cells from healthy donors [1]. In vivo, the role of endogenous B cells in cancer progression is not completely clear. They can be beneficial and contribute to tumor clearance, and in this case, up-regulate B cells response might improve disease outcome; but in some types of cancers it is the opposite, as B cells may exert an immunosuppressive effect. Consequently, it is crucial to carefully monitor the B cell phenotype before and after cell infusion. Pre-treatment of B cells to boost their response ex vivo before reinfusion has been shown to promote antitumor responses and has been widely applied in B cell transfer approaches.

Besides their intrinsic properties as mentioned before, there are many other advantages for using B cells for adoptive transfer. First, they are readily available from peripheral blood or in tumor-draining lymph nodes. Second, good manufacturing practice (GMP) protocols for B cell handling in conditions suitable for clinical applications have been developed [2]. Finally, there are numerous tools for B cell modification and engineering [3]. For instance, B cells can be modified with lentiviral vectors encoding therapeutic transgenes that are inserted in host DNA and generate long-term expression of the transgenic protein. Initially, the transduction efficiency of B cells by lentiviral vectors (LVs) was relatively low, but the development of LVs pseudotyped with RD114 cat endogenous virus, baboon endogenous virus or measles virus glycoproteins has tremendously increased transduction efficiency [4,5,6]. The expansion of genome editing techniques has also benefited B cell engineering. Of note, the Crispr/Cas9 system has been implemented in many studies to create genome modifications via gene knock-out, knock-in or mutations in B cells. Procedures are now available to routinely edit the genome of B cells with the clustered regularly interspaced short palindromic repeats/crispr associated protein 9 (CRISPR/Cas9) system and to expand them ex vivo prior to reinfusion, although editing efficiency must be improved [7].

So far, clinical trials of adoptive B cell transfer in human remain limited [8]. The first clinical trial dates back to the 2000s, when autologous leukemic B cells isolated from chronic lymphocytic leukemia (CLL) patients were transduced to express the human CD40L or IL-2 gene prior to cell reinfusion. Following this first trial, two other studies were launched, relying on a similar principle: allogenic B cells from healthy donors were fused electrically with autologous tumor cells and injected into patients [9,10]. These trials led to mitigated results with remission for some patients who generated antitumor response, more particularly T cell responses following vaccination with fused cells, but with no effect for other patients enrolled in the studies. Only minor side effects were detected. More recently, a first-in-man phase I/IIa clinical trial was started in transplanted patients to evaluate the safety and tolerability of B cells transferred concomitantly to hematopoietic stem cell (HSC) infusion, aiming to counteract immune defects and rapidly confer humoral immune protection [11]. No adverse events, especially graft versus host disease, were reported (conference communication mentioned in [12]).

Here, we review the adoptive transfer assays of B cells, ex vivo-modified or not prior to re-infusion, which have been performed to cure cancers in preclinical mouse models and that might be transposed to humans in the coming years.

## 2. Adoptive Cell Therapies with B Cells Loaded with Tumor Antigens

A critical question when performing adoptive transfer with B cells loaded with tumor antigens is to define which peptide(s) must be used (Table 1). In fact, B cells should interact with antigen-specific T cells to promote functional immune responses. However, tumor-specific antigens are rarely known and often vary among patients. As an alternative, tumor-associated antigens (TAAs) that are over-expressed in cancer cells but also expressed at a lower level in healthy cells—thus not tumor-specific —are commonly used. TAAs can vary in their structure and origin; they include “cluster of differentiation” antigens, vascular targets and growth factors. Thus, depending on the cancer type and on the patient, the antigens selected for loading can differ widely. Tumor antigens for loading should be chosen carefully, both to reduce adverse events and to maximize immunogenicity, as some regions are more potent in inducing immune responses. In addition, several peptides can be loaded simultaneously to further enhance antitumor immune efficacy.

Although some studies attempted to perform antigen loading on B cells directly in vivo, for instance by fusion of an anti-CD19 mini-antibody to promote recognition and presentation [26], ex vivo antigen loading is the most commonly used method. Aside from being safer, it also allows activation of B cells prior to adoptive transfer. In the absence of costimulatory molecules on B cells, T cells cannot be activated and become anergic, which can worsen the disease. Thus, the expression of CD40 on B cells has been shown to be necessary for T proliferation and activation [27]. Conversely, ex vivo stimulation of B cells through the CD40/CD40L pathway by cross-linking molecules also promotes polyclonal activation and cell proliferation, leading to the expression of other stimulatory molecules, such as major histocompatibility complex II (MCH-II) and CD80/86 at the B cell surface [16]. The expression of these costimulatory molecules can enhance T cell activation. Moreover, ex vivo-activated B cells are resistant to immunoconversion toward a tolerogenic state, which can be triggered by the tumor microenvironment [16]. These cells may also have an increased ability to penetrate tumors [28]. Of importance for cancer applications, CD40-activated B cells from patients proliferate and prime antitumor T cell responses in a manner comparable to those derived from healthy subjects [29]. As mentioned above, in parallel to ex vivo activation by defined culture conditions that increase their activation potential, B cells are also loaded with tumor antigens, either directly as peptides or encoded by RNA, DNA or viral vectors to compel their presentation (Table 1).

### 2.1. B Cells Loaded with Tumor Antigen Peptides

First, B cells can be loaded with tumor peptides. These peptides can be collected directly by tumor dissociation and lysis or can be synthesized in vitro. For instance, tumor peptide loading on CD40-activated B cells resulted in effective induction of antigen-specific T cell responses after antigen presentation [30,31]. In the mouse model of B16.F10 melanoma and E.G7 lymphomas, transfer of such modified cells was sufficient to drive a significant decrease in tumor growth and even complete eradication of tumor cells in some studies [20,21].

### 2.2. B Cells Modification with RNA Encoding Tumor Antigen

Compared to peptide loading, RNA transfer has several advantages to induce presentation of tumor antigens on B cells, among which are safety, cost and relatively easy handling. RNAs can be introduced into cells by transfection or electroporation, delivery methods that are compatible with good B cell viability. Electroporation of B cells with mRNAs expressing carcinoembryonic antigens prior to cell transfer in a colorectal cancer model efficiently generated cellular and humoral antitumor immune response, reducing tumor growth [22]. Similarly, an antigen-specific T cell response (IFNγ and lytic enzymes secretion) was induced following stimulation with a melanoma-associated antigen recognized by T cells (MART-1: melanoma antigen recognized by T cells 1) mRNA-loaded CD40-activated B cells [32].

### 2.3. B Cells Engineered with DNA Encoding Tumor Antigens

Antigen expression can also be induced via a naked DNA sequence, which can be inserted into B cells either spontaneously after addition to the culture media or by electroporation. DNA-encoded antigen-primed B cells are efficient antigen-presenting cells and can induce antigen-specific IFNγ and IL-2 release by CD8 T cells. In the long term, CD8 T cells generated following B cell infusion persist, and can generate a long-lasting protective immunity [33,34]. Moreover, modification of B cells with DNA has been shown to up-regulate costimulatory molecules such as CD86, which might further enhance therapeutic efficacy [35]. Such approaches have been applied in cancer treatment. For instance, uptake of DNA encoding the tumor-associated antigen SSX2 (synovial sarcoma X 2) by B cells resulted in the induction of T cell response in vitro [35].

### 2.4. B Cells Transduced with Viral Vectors Encoding Tumor Antigens

As DNA uptake by B cells is not very efficient, several viral vectors have been developed to transfer and express specific DNA sequences into these cells. Some viral vectors insert the DNA into the host genome (i.e., integrative vectors, such as lentivirus-based vectors) or remain episomal (i.e., non-integrative vectors, such as adenovirus-based vectors). For instance, adenoviral vectors were used to introduce sequence coding of the epitopes of the Human Epidermal Growth Factor 2 (HER-2) into B cells, which led to the generation of antibodies against HER-2 and of a cytotoxic T lymphocyte (CTL) immune response. Following infusion of these cells in vivo, both reduction in tumor growth and improved survival were observed [25]. Further enhancing adenovirus efficacy, modification of the structure of fiber virion surface protein was performed to promote B cell transduction. Encoding of the HER-2 TAA by such modified vectors drove strong-antigen specific humoral and cellular responses after B cell transduction, which prevented growth of established tumors [36]. Alternatively, addition of the CD40 ligand ectodomain on the surface of adenovirus vectors that carried the HER-2/neu or human papillomavirus 16 (HPV16) E6/E7 genes allowed the delivery of these tumor antigens while concomitantly activating B cells. This activation led to enhanced CTL response and significant inhibition of tumor growth [23]. Lentiviral vectors (LVs) have also been applied to induce the expression and presentation of tumor antigens by B cells. For instance, the delivery by lentiviral vectors of melanoma-associated antigen tyrosinase into B cells induced the stimulation of antigen-specific patient T cells [37]. Of note, OVA-transgenic B cells induced higher CTL response and higher tumor growth reduction in an EG-7 thymoma tumor model expressing ovalbumin (OVA) protein, as compared to B cells loaded with OVA peptides [17]. This suggests that prolonged antigen expression and then presentation may increase treatment efficacy, making viral vectors good candidates for tumor antigen delivery.

**Figure 1:**
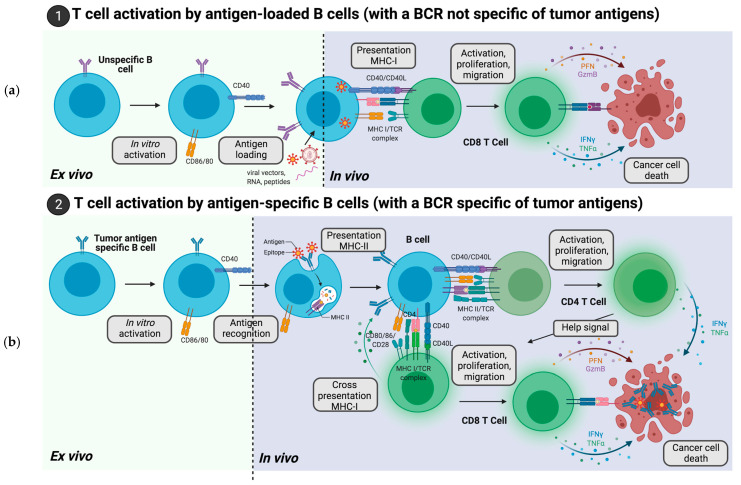
Tumor-specific T cell activation by B cells loaded with tumor antigens or with a BCR specific to tumor antigens. (**a**) B cells isolated from peripheral blood (human) or spleen (preclinical studies) are activated with a cytokine cocktail in vitro, which leads to the expression of costimulatory molecules such as CD40 and CD80/86, before being loaded ex vivo either directly with tumor peptides or with RNA or viruses encoding these peptides. This loading leads to the presentation of tumor antigens on MCH-I complexes, which interact with CD8+ T cells that are harboring a TCR specific of the presented antigen. This specific TCR/MCH-I antigen recognition combined with the CD40L/CD40 and CD28/CD80/86 interactions between, respectively, T and B cells leads to T cell activation. Once activated, the CD8+ T cells proliferate and migrate to tumor sites, where they secrete pro-inflammatory mediators (IFNg, TNFa), which boost the immune system locally, but also lysis molecules (perforin, granzyme) that directly destroy tumor cells. (**b**) Tumor-specific B cells isolated from a tumor-draining lymph node or generated by gene editing (see below) are expanded and activated in vitro (similarly to “a”) before infusion. When the tumor-specific B cells meet their target antigen in vivo, this leads to B cell activation, secretion of antibodies directed against this tumor antigen and presentation of the tumor antigen on MHC-II complexes. The presentation of tumor antigens on MCH-II leads to the activation of antigen-specific CD4+ T cells, which proliferate and provide the ‘help’ signal required for the activation of several T cell subtypes. Of note, antigen-specific CD8+ T cells can also be activated by B cells after cross presentation of tumor antigens on MCH-I complexes. In addition to the effects of activated T cells on tumor survival, the antibodies directed against tumor epitopes, secreted by B cells, are also involved in tumor clearance by triggering the activation of the complement system (CDC), the phagocytosis of tumor cells (ADCP) and the lysis of tumor cells (ADCC).

Overall, ex vivo loading of B cells with tumor antigens before infusion is possible and drives the presentation of tumor-associated peptides on MCH-I complex, which may activate antitumor CD8+ T cells (Figure 1). With this set-up, antitumor antibodies will not be directly secreted by infused B cells but rather by endogenous antigen-specific B cells after their activation by epitope-specific T cells. These T cells would have been previously activated by the transferred B cells [24]. Such strategies are mainly developed to enhance antitumoral cellular responses.

Nevertheless, it is likely that the development of B cells that encode a BCR specific for a tumor antigen might be a better alternative to the above strategies, since the target antigen would be processed more rapidly by the BCR machinery, as compared to classical antigen internalization by pinocytosis, for instance. Moreover, infused antigen-specific B cells would also secrete antibodies after activation and are more potent in inducing specific T cell response. Indeed, after internalization of the BCR/antigen complex, the tumor antigens presented on MCH-II complex or eventually MCH-I complex by cross-presentation respectively activate CD4+ or CD8+ T cells that are specific of the presented antigens (Figure 1). In contrast to the above-mentioned B cells loading strategy, such approaches may efficiently trigger cellular but also humoral responses.

## 3. Adoptive Cell Therapies of B Cells Harboring a BCR Specific for a Predetermined Tumor Antigen

### 3.1. B Cells with an Endogenous BCR Specific of Tumor Antigens

The immune systems of cancer patients have the ability to mount anticancer adaptive immune responses because B and T cell clones specific to tumor antigens circulate and are present in secondary lymphoid organs (such as the spleen or the tumor-draining lymph nodes TDLNs). However, their therapeutic effects remain limited mainly because of their restricted number, the tumor microenvironment and the immune silencing induced by cancer cells themselves. To increase their frequency, immunization with tumor antigens (e.g., OVA) has been performed in mice before isolation of OVA specific B cells with antigen tetramers. After in vitro stimulation to generate CD40+ B cells and plasma cells, these cells were transferred in mice bearing OVA+ tumors leading to antigen-specific T cell responses, with a marked decrease of tumor growth and death [18]. Paving the way for translation to humans, human tumor antigen-specific B cells collected in tumor-draining lymph nodes were able to induce antigen-specific T cell responses in vitro [18].

Indeed, due to their secondary organ function and their anatomical location close to tumors, TDLNs contain tumor-specific B cells (Table 1). B cells isolated from TDLNs of 4T1 tumor-bearing mice (a spontaneous metastasis model from 4T1 tumor cells implanted in the mammary fat pad) have been shown to be activated in the presence of irradiated tumor cells and to secrete IgG and IFNg in large amounts after ex vivo coculture [14]. Moreover, perforin is also secreted by TDLN-derived B cells and drives tumor cell death, which can also be mediated by the Fas/FasL pathway [13]. B cells from TDLNs express chemokine receptors that allow them to migrate to target areas by chemotactism [13]. After in vivo infusion of these TDLN-derived B cells in the 4T1 tumor mouse model, an antitumor immunity was triggered and spontaneous lung metastases were reduced, notably through antigen-specific T cell cytotoxic responses (cytokines, lytic enzymes) and antitumor antibody secretion [13,15].

Alternatively, tumor-infiltrating lymphocytes have gained interest for adoptive cell transfer therapies. Tumor-infiltrating lymphocytes (TILs) are mainly T cells and can be collected by tumor excision. Expansion and ex vivo activation of these cells lead to the capacity to infuse a greater number of cells and in a phenotype that is more prone to slow tumor progression [38]. Such approaches have mainly been performed with T cell-derived TILs, although as B cells also infiltrate certain tumors, such as breast cancers, similar strategies might be encompassed with B cell-derived TILs.

However, this kind of approach remains limited by the amount of tumor-specific B cells that can be retrieved. Techniques to sort antigen-specific B cells have been developed, notably by coupling these antigens to a fluorophore or to fluorescent beads [39], but this does not increase the number of retrieved cells. Thus, toward the goal of increasing antigen-specific B cells, a culture system was developed to expand antigen-specific B cells specifically. It relies on the culture of B cells, with a feeder cell line expressing activating cytokines as well as the desired antigen along with Fas ligand (FasL) [19]. For cultured B cells expressing a BCR specific to the harbored antigen, signaling by the BCR cascade can rescue cells from FasL-induced cell death, while unspecific B cells die. Nevertheless, the numbers of antigen-specific B cells that are available remain low. To counteract this issue, many preclinical proof-of-concept studies relied on the transfer of B cells from BCR transgenic mouse models with engineered known BCR specificities, i.e., that express BCRs directed against a given antigen to obtain antigen-specific cells in high amount (Appendix A). Alternatively, one promising approach for reaching high numbers of antigen-specific B cells and redirecting them against tumors is to introduce the sequence of a new tumor antigen BCR by viral vectors or by gene editing in B cells, which may hijack their endogenous specificity.

### 3.2. B Cells Engineered to Express a BCR Specific of Tumor Antigens

Hijacking the specificity of the endogenous BCR to confer new specificities to B cells is possible by genetic-reprogramming vectors. The expression of a new immunoglobulin by B cells can be induced either by directly modifying B cells themselves or by engineering hematopoietic stem cells, ensuring that all B cells descending from a modified stem cell after differentiation will harbor the modification. For instance, lentiviral vectors carrying the whole sequence of a new antibody were used to transduce B cells or, alternatively, hematopoietic stem cells, leading to the expression of membranous or secreted transgenic antibodies by modified cells in vivo [40,41,42]. Going further, by engineering the transgene sequence, a lentiviral vector allowing either the expression of a membrane-anchored antibody (BCR) or a secreted antibody depending on the B cell maturation status was successfully developed [42]. This system is physiologically regulated and does not depend on externally provided signals. However, with such reprogramming viral vectors, the endogenous BCR is still expressed, which might lead to light- and heavy-chain chimeras and to signaling cross-talks. Indeed, when the ectopic BCR recognizes its target, it leads to B cell differentiation and consequently to the secretion of the endogenous antibody, which may trigger adverse events. Moreover, as lentiviral vectors integrate randomly into host genomic regions and potentially disrupt crucial genes, their use raises additional safety issues for clinical applications.

**Figure 2:**
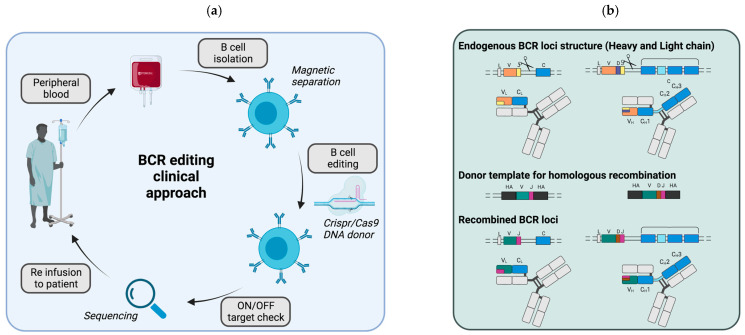

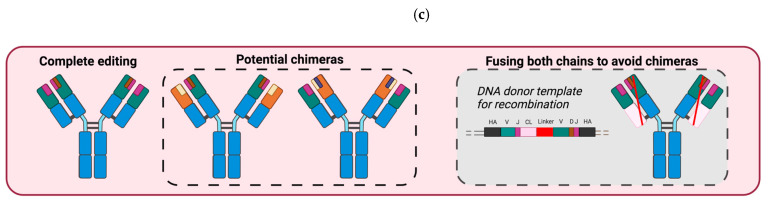
Editing of the endogenous BCR loci to modify B cell antigenic specificity. (**a**) Potential clinical set-up for therapies involving BCR editing. B cells are isolated by magnetic separation from the peripheral blood of patients (or of a compatible healthy donor, allogenic graft) before ex vivo editing. For editing, the CRISPR/Cas9 is used to introduce double strand breaks that are then repaired by homologous recombination if a repair template is provided by adeno-associated viral vectors. Prior to cell reinfusion in the patient, a safety step should be added to check both the editing and the OFF target mutations. (**b**) BCR loci before and after editing. Three distinct genes encode the BCR: one for the heavy chain (right) and two for the light chain (kappa or lambda, left). Of note, only one light chain gene is expressed per cell, either kappa or lambda. The recognition specificity of the BCR is determined both by the combination of a variable (V), a diversity (D) and a joining (J) region on the heavy chain and by the combination of a variable (V) and a joining (J) region on the light chain (top). The constant regions (blue) are not involved in antigen recognition directly but in the triggering of effector responses. Initially, the first constant domains (u and d) are expressed leading the IgM or IgD antibodies. After B cell activation by a process called isotypic class switching, these first constant domains can be genetically excised and the following constant domains are expressed leading to either IgG, IgA or IgE antibodies. Most editing strategies are aimed at introducing a new VDJ combination into the heavy chain locus just before the constant domains in order to change the BCR specificity. This insertion can be performed after introduction of double strand break with the CRISPR/Cas9 system into the heavy chain locus, and the new VDJ combination is inserted using a donor template that harbors homologous arms for recombination on each side of the VDJ sequence to introduce. Similarly, the light chain loci can also be edited concomitantly to modify both chains. However, this often results in poor editing rates, due to low recombination efficiency. To counteract this issue, the whole light chain domain can be inserted along with the VDJ heavy chain domain into the heavy chain locus. (**c**) BCR chain chimeras can arise if the editing is incomplete. If only one chain is edited, it will pair with the endogenous version of the other chain, which can modify the specificity of the BCR and might lead to autoimmune reaction. To avoid this mispairing, one study physically linked the light and heavy chains in order to force proper pairing (bottom right). In this case, the donor template contains the whole light chain and the VDJ domain of the heavy chain with a linker in between. This cassette is flanked by homologous arms for insertion into the heavy chain locus just before the first constant domain by homologous recombination.

In this context, the CRISPR/Cas9 system appears to be a good strategy to modify the endogenous BCR loci (Figure 2a). The aim is to drive the integration of the variable regions of a new monoclonal antibody directed against a target antigen at a defined genomic site—preferentially the endogenous locus itself, where the cleavage by the Cas9 protein is induced. The CRISPR/Cas9 system consists in different components: a guide RNA and a Cas9 protease that cleaves double-stranded DNA at specific sites. Double-strand breaks can then be repaired by two different repair mechanisms: the error-prone process of non-homologous end-joining (NHEJ), which repairs DNA by random nucleotide addition, or through the error-free homology-directed repair (HDR), which requires a donor DNA template to repair DNA by homologous recombination. Paving the way toward this goal, the CRISPR/Cas9 system was recently used to generate a CD19-knockout in B cells [43]. However, only one locus encodes CD19, while immunoglobulins are encoded by three distinct loci, which renders the editing even more complex (Figure 2b). Indeed, the heavy chain locus located on chromosome 14 is composed of four different region types (variable (V), diversity, (D) joining (J) and constant (C)), whereas the κ and λ light chains (kappa and lambda) are located on chromosomes 2 and 22, and comprise only the variable, joining and constant regions.

During their development, B cells undergo genetic rearrangements generating random combinations of VDJ regions and VJ in the heavy and light loci, respectively, thus creating an almost unique combination for each newly generated B cell. Although some combinations are more frequent than others, this sequence diversity makes it difficult to design guide RNAs capable of cutting all B cells in their variable regions. Of note, one study succeeded in efficient cleavage of the VDJ regions of the BCR heavy chain, by designing guide RNAs that bound specifically to the V and J regions [44]. The double-strand breaks subsequently allowed the introduction of a new variable heavy region by homologous recombination, using a donor DNA template delivered by adenovirus-associated virus (AAV) vectors [44]. Moreover, in addition to the editing of the heavy chain locus, a new light chain should also be added to completely change the immunoglobulin specificity. This chain can be inserted into the heavy chain locus along with the heavy chain variable sequence [45] as well as in one of the light chain loci [46]. For instance, modification of both the heavy and kappa light chains was achieved after cutting in the variable domains, leading to the expression of a brand-new immunoglobulin [46].

To counteract this difficulty in targeting full combinatorial diversity, the variable regions can be inserted into the intronic region of the heavy locus between the last J-domain and the µ-constant region that are common to all B cells [45]. However, insertion into this site, which is far away from the endogenous immunoglobulin promoter, requires the addition of an ectopic promoter to control the expression of the edited sequence. However, replacement of the endogenous variable regions by new regions allows full exploitation of the endogenous promoter to regulate the edited immunoglobulin gene, which is more physiological.

During B cell maturation, immunoglobulins undergo somatic hypermutations to increase antibody affinity and isotypic class switching. This last process consists of genetic rearrangements on the heavy chain locus after cleavage by the activation-induced cytidine deaminase (AID) enzyme to remove proximal constant exons and keep distal constant exons (such as γ1, γ3, α1 leading to IgG1, IgG3 and IgA1 respectively). These two mechanisms contribute to the enhancement of the humoral response and may also occur on the modified BCR loci. Interestingly, class switching following the integration of variable heavy chain into the IgH locus with the CRISPR/Cas9 system was observed, as antibodies against the target antigen of several isotypes (IgM, IgG and IgA) were detected in the serum of mice infused with edited B cells [44,47]. In addition to the natural regulation process of class commutation, the CRISPR/Cas9 system has also been used to compel isotypic class commutation from IgM to IgG by the designing of guide RNAs, deleting the constant-µ/regions in the heavy chain locus [48]. After recognition of the antigen, B cells are activated and a memory B cell compartment is also generated, providing protection on the long term. Recently, a study showed that edited B cells can be activated and differentiate into memory and plasma cells after immunization in vivo, thus demonstrating that B cells retain their functionalities after editing [47].

Nevertheless, such BCR editing approaches have been limited both by the CRISPR/Cas9 system itself, e.g., by off-target integrations or mutations, and by its specific application for BCR editing, which imposes more constraints. Indeed, the CRISPR/Cas9 technology in B cells exhibited a low editing efficiency, owing to low cleavage efficiency for some studies (26–55% cleavage) but also to low recombination efficiency (0.2–30% homology directed repair (HDR) efficiency) [44,45]. Furthermore, since such studies attempted to modify two of the BCR encoding loci (i.e., one light and the heavy V domains), statistically, the efficiency of dual editing was even lower. However, to obtain a fully active antibody, the modified heavy chain must be specifically linked with the modified light chain, thus both loci need to be edited. Indeed, the modification of either the heavy or the light chain alone would produce chimeric antibodies with a modified heavy chain and an endogenous light chain or vice-versa (Figure 2c). These chimeric immunoglobulins can therefore have a different specificity and may even recognize self-antigens, thus leading to induction of autoimmune reactions. For instance, modification of only the heavy chain in embryonic stem cells blocked B cell differentiation, suggesting that self-reactive clones were deleted in the bone marrow by tolerance mechanisms [49]. Finally, besides the requirement of editing both BCR loci, their two alleles should also be edited to avoid chimeras. Due to a mechanism called allelic exclusion, only one allele coding the immunoglobulin chains is expressed (productive allele). Consequently, if the editing is performed on the productive allele, only the modified chain will be expressed, while if the editing is performed only on the non-productive allele, both the edited and the endogenous chains will be expressed. To circumvent this chain-pairing issue, a linker between the light chain and the variable heavy chain was added on the DNA template to force the correct pairing between the modified light and heavy chains after insertion in the heavy chain locus (Figure 2c) [45].

Although most studies focused on ex vivo BCR editing, one study recently attempted to perform BCR editing directly in vivo using two adenoviral vectors, encoding respectively the CRISPR/Cas9 system and the donor template [50]. Following vector infusion, engineered antibodies were detected in mouse sera, showing the feasibility of this kind of approach. While this strategy seems highly attractive, owing to its putative simplicity, some safety issues remain to be addressed before implementation in humans. Indeed, only B cells should express the immunoglobulin as other cell types might not allow its correct folding. Thus, to overcome this hurdle, a B cell specific promoter was used in the adenoviral vector to control the immunoglobulin expression. Nevertheless, modification of other cell types increases the risk of off target mutations, which may trigger serious side effects such as cancers. This field will surely benefit from novel, more specific vectors that can transduce defined cell types in vivo.

Overall, the editing BCR strategies developed so far are aimed at reprogramming the BCR specificity against viruses, but have not yet been applied to treat cancers. In the coming years, similar approaches against cancers may be launched by replacing the immunoglobulins’ variable regions with those of monoclonal antibodies directed against tumor-associated antigens.

## 4. Adoptive Cell Therapies of B Cells with Enhanced IMMUNO-Regulatory Properties

Besides compelling antigen presentation at the B cell surface, the engineering of B cells can also enhance treatment efficacy by playing on the *de novo* expression of costimulatory ligands, which can interact with other immune cells or can secrete immuno-regulatory soluble mediators.

### 4.1. Modulation of CoStimulatory Immune Cell Ligands

The concept of modifying B cells to promote their stimulatory potential is not recent, as in 1998, an adenoviral vector was used to trigger the expression of a functional ligand for CD40 in leukemia B cells [51]. This engineering rescued the antigen presentation capacity of leukemia B cells, which led to the induction of specific T cells [51]. Following this study, comparable results were obtained after modification of leukemic B cells using lentiviral vectors encoding costimulatory genes, such as CD80 or granulocyte-macrophage colony-stimulating factor (GM-CSF) [52]. Moving one step beyond, lentiviral vectors encoding costimulatory ligands, such as CD40L, CD70, OX40L, or 4-1BBL, have been used in pairs to further enhance B cell costimulation capacity, resulting in potent cytotoxic activities and increased survival rates in a B16 melanoma mouse model [53]. Other delivery methods to induce the expression of costimulatory molecules by B cells have been employed, such as RNA electroporation. Electroporation of multiple messenger RNAs encoding costimulatory molecules (OX40L and 4-1BBL), cytokines (IL-12p35 and IL-12p40) and antigens in B cells induced IFNγ secretion by antigen-specific CD8 T cells in vitro [24].

Although modulation of costimulatory molecules at the B cell surface designed to improve interactions with CD8 or CD4 T cells has been the focus of many studies, B cells can also interact with other immune cells, such as Natural Killer T (NKT) cells. NKT cells are of particular interest in cancers, since they are implicated in the reversal of the immunosuppressive state induced in tumors. Thus, the NKT cell ligand, α-galactosylceramide (aGalCer) was loaded into B cells and then presented to CD1d molecules [24]. This presentation increased activation of NKT cells and contributed to the boost in immune responses [24].

### 4.2. Modulation of Immuno-Regulatory Molecules Secretion or Recognition

B cells can also naturally secrete immuno-regulatory molecules, which vary in activation and differentiation status. Thus, it is possible to skew their maturation by genome editing in order to infuse cells more prone to exerting the desired therapeutic effect. For instance, knock-out of specific genes generates more or less differentiated plasma cells, depending on the target genes [54]. While such techniques remain quite sophisticated, an easier approach consists of infusing the B cell subtype adapted for therapeutic purposes after sorting. For instance, long-lived plasma cells are specialized in antibody secretion, and reside in the bone marrow for a lifetime. In a study exploiting their specific secretion and long-term properties, these cells were genetically engineered with the CRISPR/Cas9 technique to insert an anti-PD-1 antibody transgene into the *GAPDH* locus. This transgene allowed a *de novo* persistent secretion of antibodies, which inhibited human melanoma growth in a xenograft-tumor model notably mediated by antitumor T cell responses [55]. Of note, the CRISPR/Cas9 system can also be applied to disrupt inhibitory checkpoint genes that may impair B cell activation after reinfusion of modified cells and interaction with tumor cells. The secretion of pro-inflammatory cytokines by infused edited B cells may synergically contribute to counteracting the immunosuppressive tumor environment. Paving the way towards this goal, the secretion of BAFF by infused B cells was induced following gene insertion in the CCR5 locus with the CRISPR/Cas9 system [54]. Conversely, the secretion of immunosuppressive cytokines that are involved in the generation of an anti-inflammatory state can be blocked. For instance, the transfer of TDLN B cells deficient in IL-10 in 4T1-tumor bearing mice significantly enhanced antitumor immunity [28].

Combined to the induction of a pro-activating tumor microenvironment, molecules that facilitate the recruitment of B cells at the tumor site (particularly for solid tumors) could also be expressed. For instance, T cells have been forced to express a chemokine receptor C-X-C type 6 to enhance their homing [56], paving the way for translation to B cells.

## 5. Synthetic Circuit to Control Cellular Responses

### 5.1. General Principle

As previously described, several immune cells can be engineered to enhance their recognition or effector properties. Taking advantage of the above-mentioned tools to engineer such cells, the synthetic immunology field is seeking to completely reprogram cell networks with artificial gene circuits. These gene circuits are designed to boost the sensing capacities and responses of modified cells, so that depending on the local environment, these engineered cells may modulate their behavior. The ultimate goal of such approaches is to trigger therapeutic immune effectors with a higher efficacy than endogenous responses with both a spatial and temporal regulation by internal cues. Indeed, these two levels of regulation are expected to maximize treatment efficacy while reducing side effects. In cancer applications, the challenge is to specifically target tumor tissue while sparing healthy cells, which can be achieved with synthetic immunology therapies. Moreover, due to the long-term persistence of immune cells, such approaches hold great promise for the generation of efficient immune responses in cancer patients.

Adaptive immune cells, and more particularly T cells, have been investigated for synthetic immunology therapies due to their endogenous properties (Appendix B). Indeed, T cells as well as B cells freely circulate in the body by way of patrol, and have the ability to infiltrate tissues [57]. Moreover, their natural function is to sense deviations from the homeostatic state and to trigger various responses, including molecule secretion and communication with other cells, in order to restore homeostasis. New effector functions of immune cells, as well as new recognition capacities, can be conferred alone or simultaneously by genetic circuits. The most well-known example of such a reprogramming is chimeric antigen receptor (CAR) T cells, which have been approved by the European Medicines Agency (EMA) to treat B cell malignancies [58]. Several generations of CAR constructs have been designed to target tumor antigens using ScFv fragments and to induce T cell activation after signaling through intracellular domains fused to the recognition fragment. Interestingly, in the fourth generation of CAR T cells, aside from the endogenous activation of the modified T cells, the regulated secretion of therapeutic molecules, such as cytokines, have also been implemented. The gene encoding the therapeutic molecule under the control of a nuclear factor of activated T cells (NFAT) sensitive promoter (activated by the CAR signaling pathway) is inserted into T cells along with the CAR transgene [59]. Consequently, upon the binding of tumor antigens on the CAR, the NFAT sensitive promoter is activated and the cytokine is secreted. Notably, this allows the stimulation of other immune cells that infiltrate tumors, but also helps to counteract the globally immunosuppressive tumor microenvironment, which inhibits the efficacy of the infused cells [59,60].

**Figure 3:**
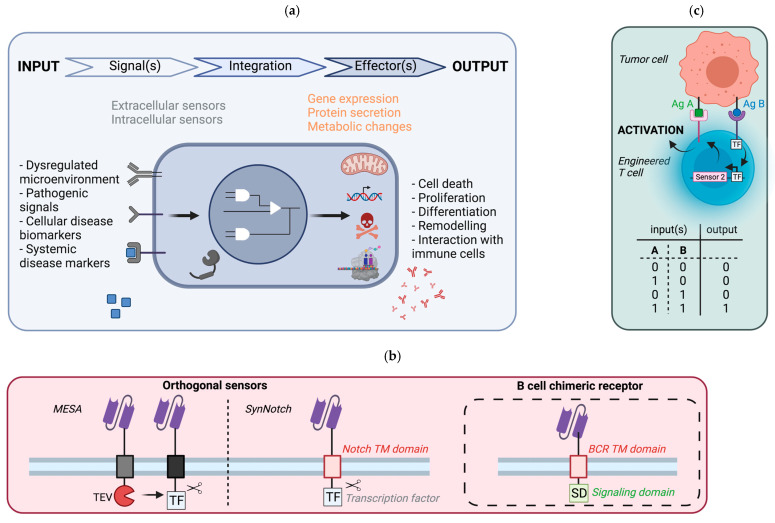
Synthetic immunology approaches applicable to B cell therapies. (**a**) General principle of synthetic regulatory networks. A disruption of homeostasis, such as a local dysregulation, the presence of pathogenic motifs or disease biomarkers, can be recognized by dedicated sensors. These sensors transduce the signals by signaling pathways. Depending on the circuit architecture, several signals may be computed to generate different responses depending on the signal strength and/or the combination of signals detected (gene expression, metabolic changes). (**b**) Chimeric synthetic sensors transposable for the regulation of synthetic networks in B cells. The MESA approach relies on two sensors, both having ScFv domains for target recognition and the CD28 transmembrane domain, but differing in their intracellular parts: one contains the TEV protease, while the other harbors a TEV cleavage site before an ectopic transcription factor. Upon the binding of the target antigen, the transcription factor is released by cleavage on the TEV site. Similarly, the SynNotch sensor is composed of ScFv fragments for the recognition of the target, and fused to the transmembrane domain of the Notch receptors linked to an ectopic transcription factor (middle). Upon the binding of the antigen, a mechanical force triggers the release of the orthogonal transcription factor (the Notch receptor is naturally cleaved after the binding with its ligand Delta), which activates the transcription of a transgene inserted under the control of a promoter sensitive to this transcription factor (ectopically provided). Chimeric BCR sensors combine the extracellular ScFv fragments (for recognition) and the BCR transmembrane regions fused to the intracellular of the Igb (CD79b) signaling domain (right). (**c**) AND-gate circuit regulation. Complex circuits with Boolean gates have been developed to refine the response depending on several input cues. An AND-gate has been developed to trigger T cell activation only if two tumor antigens are detected. When the first sensor, constitutively expressed, recognizes the antigen A, it triggers the expression of the second sensor that recognizes the antigen B, and this second recognition leads to T cell activation.

Several components must be integrated (Figure 3a) in order to obtain a fully functional synthetic circuit. First, an ‘input’ signal, ideally specific to the targeted disease, must be chosen. This input can be internal, and in this case, the system will be autonomous (for example, a tumor antigen), which is the long-term objective. Conversely, this input signal can also be external (for example light or pressure), and therefore must be provided by the user/patient himself [61,62]. Specific ‘sensors,’ or ‘receptors’, must be implemented to recognize the defined input signal. Although these sensors can be intracellular, most of the sensors developed so far are anchored at the membrane. Once the sensor detects the input signal, it initiates a transducing cascade (phosphorylation, translocation of transcription factors) that integrates the signal type and strength and generates defined responses. This transducing cascade can either be encoded genetically within the synthetic circuit (orthogonal) or exploit the endogenous signaling pathways (non-orthogonal). Of note, several sensors can be multiplexed to sense multiple input signals and modulate their functions, depending on the input combination. After signal integration and processing, various ‘effector’ functions can be triggered, such as metabolic changes or gene activation, leading to different outputs such as proliferation, cell death or molecule secretion [57].

### 5.2. Potential Implementation in B Cells

A crucial component of synthetic circuits is the sensor of the signal specific to the disease. For internal physiological signals, the range of receptors that can be applied is extensive. Natural receptors, either endogenous or ectopically expressed, can be exploited, although for these kinds of sensors, the corresponding transducing cascades would not be orthogonal to cell signaling pathways, and may lead to deleterious cross-talks. For instance, edited BCRs as above-described fall into this category. Alternatively, the implemented receptors can be fully synthetic [63,64]. Depending on their design, they can be orthogonal or not orthogonal to cell signaling, and their multiplexing may be possible. For example, two reprogramming platforms have been engineered and rely on fully chimeric synthetic sensors that are orthogonal and that can be multiplexed: namely, the SynNotch (Synthetic Notch) and the MESA (Modular Extracellular Sensor Architecture) platforms (Figure 3b) [65,66,67]. Although many synthetic sensors that are in theory compatible for expression in B cells have been constructed, only a few have been tested in B cells. Among them, the CBCR (chimeric BCR) has been introduced with the CRISPR/Cas9 system into B cells. This receptor is composed of an ScFv (for recognition of the target antigen—the signal), a spacer region that includes StrepTag motifs (for detection of the sensor) and a transmembrane domain fused to the intracellular domain of CD79b (for induction of B cell maturation and activation—the effector) [68]. Following gene editing, the CBCR receptor was expressed in primary B cells and shown to bind to its target antigen. Nevertheless, its signaling capacity still needs to be addressed. Overall, many completely programmable sensors, both in terms of sensing and transduction pathway, have been designed; however, their application in B cells remains limited. Further studies are required to fully address their compatibility and functionality in B cells, which will then open the path for new B cell-based therapies.

Although promising, synthetic immunotherapy also presents significant challenges that limit its widespread use for cancer treatment. Indeed, toxicity due to off-target interactions or cytokine storms remain concerning safety issues. To address these safety issues, several approaches have been undertaken. The most straightforward is to insert suicide genes, leading to cell death after the administration of defined molecules [69]. Alternatively, more refined ways to improve safety, control and precision based on the Boolean logic have emerged [70,71]. For instance, the detection of a pathological state can be improved by the dual recognition of two inducing signals, both required to trigger effector outputs: this is an AND-gate (Figure 3c) [63,65]. As healthy cells can also express tumor-associated antigens, adding such gates may reduce the recognition of healthy cells as pathological cells. In addition, safety switches can also be implemented through feedback loops that can be positive or negative. Positive feedback loops can enhance treatment efficacy after initiation of the response [72]. Conversely, negative feedback loops ensure that the system stops once the danger signals are absent, avoiding unnecessary effector functions that may lead to side effects.

While many significant advances have been achieved, this field is still hampered by the limited number of genetic modifications that can be performed. Indeed, the maximal transgene size that can be packaged and delivered with lentiviral vectors is around 10kb, which allows the encoding of only a few genes [57]. Co-infection with multiple viruses may be a solution, but this can cause cell variability and heterogeneity, hence decreased reproducibility, which is undesirable for clinical applications. Moreover, editing efficiency with gene editing systems remains low in primary B cells (around 10%). Progress in gene engineering techniques in the coming years will no doubt benefit future synthetic immunology strategies. Specific selection and amplification of modified therapeutic cells could also be envisioned to counterbalance low editing efficiency.

## 6. Conclusions and Perspectives

During the previous decade, T cells for adoptive cell transfer cancer therapies were a primary focus because they are highly cytotoxic and easily expandable. This led to great successes, including CAR T cells. Recently, B cells have regained interest in the research involving adoptive cell transfer as a cure for cancers. Here, we reviewed the main approaches that involve adoptive transfer of B cells to cure cancers. These approaches often exploit the endogenous properties of B cells, such as antigen presentation, either by forcing the presentation of antigens through external loading, or by amplification B cells with a BCR specific to tumor antigen (sorting or editing). The infused B cells can then interact with and activate other immune key players, such as T cells, strengthening the antitumoral response. Although the preclinical data hold great promise, many questions remain to be addressed before translation to human disease treatment.

First, the number of cells needed to transfer in order to trigger a therapeutic effect is not clear. Indeed, it depends on cell engraftment and cell survival. If the cells are modified to increase their recognition or effector properties, the modification efficiency, which will not be 100% (unless modified cells can be sorted), should also be taken into account. Moreover, the required number of infused B cells also depends on the application, namely antigen presentation or antibody secretion. A partial answer to this problem is that to elicit a humoral response after an antigenic stimulation, 1x10^4^ monoclonal NP-reactive B1-8 B cells are sufficient to give rise to a *bona fide* humoral anti-NP response after transfer into AM 14 HEL-reactive BCR mice [73]. This number is in line with the amount of pre-immune B cells that are specific for Phycoerythrin in the lymph nodes and spleen of naïve C57Bl/6 mice (4 × 10^3^ to 2 × 10^4^) [74].

In addition, only 10^2^ transgenic B cells loaded with DNA to present tumor antigens are sufficient to induce antitumor CD4 and CD8 T cells in mice [34], a significantly smaller number which may therefore be more easily transposable to humans. However, when these numbers are rescaled to human body weight, the number of cells required for engraftment is very large compared to the number of B cells that can be harvested from peripheral blood or from draining lymph nodes. This may become critical if the graft is not allogenic but autologous, as in the case of cancer patients who are often lymphocytopenic, due to the disease itself or to chemotherapy. Moreover, there is often variability among patient-derived B cells; thus, using well-defined allogenic B cell batches may be better in terms of both quality and safety. Protocols to expand human immune cells ex vivo in GMP conditions have been developed for clinical application, mainly for T cells, but in the previous decades, some procedures have also been developed for B cell expansion, paving the way for clinical translation [2].

Nevertheless, before translation, the route of cell infusion must also be determined. The two main routes of injection for such applications are the intravenous or subcutaneous routes. Following subcutaneous injection, the cells must migrate from the injection site to lymphoid organs, where they present antigens to T cells and activate them. Although some studies did not show any therapeutic difference whatever the route of injection tested [53], the intravenous route was s the most widely used, as B cells have a poor ability to migrate to lymphoid organs after subcutaneous injection, while B cells transferred intravenously readily migrate to secondary lymphoid organs [24].

After reaching their target sites, namely secondary lymphoid organs or tumors, infused B cells should be able to exert their therapeutic functions. One potential danger is that B cells can switch from anti- to pro-tumor phenotype under the influence of the tumor microenvironment. Genetic safety switches can be added to engineered B cells to kill the transferred cells in case of adverse events. Infused cell effects should also be carefully monitored in order to avoid worsening the disease [75], but this remains complicated because of variability among tumor types and also among patients. For instance, such approaches could work better in immunocompetent individuals who can mount tumor-specific immune responses. To overcome the locally immunosuppressive microenvironment and to enhance treatment efficacy, the immune response can be boosted either directly by addition of stimulatory cytokines such as IL-2 [13], or indirectly by shutting off immunosuppressive molecules. For instance, editing in B cells can be multiplexed to trigger higher immune responses by knock-down of immunosuppressive genes such as IL-10 [28] or PD1 (as already performed in T cells [76]). The cotransfer of T cells along with B cells might also increase B cell engraftment and stimulation, and thus enhance tumor depletion, as compared to the transfer of B cells or T cells alone [15]. Furthermore, adoptive B cell transfer can also be combined with other conventional cancer therapies, such as chemotherapies or radiotherapies, in order to increase tumor killing.

To facilitate translation to humans, new preclinical models of valuable importance have been developed [77]. For instance, humanized mice with a human immune system (HIS) have been engineered by engraftment of cord blood cells into immunodeficient mice. Of note, successful adoptive transfer of GFP marker-transduced B cells between HIS mice was recently performed in our lab, opening the path for the testing of new therapies involving B cell transfer in a context more closely mimicking humans. Adoptive B cell transfer may also benefit from other fields, such as gene engineering or nanotechnologies. Moreover, as B cells are involved in several immune-related pathologies, such as infections and autoimmune diseases, strategies involving B cell transfer have been tested as a permanent cure with encouraging results. In the coming years, progress for a given therapeutic application will likely benefit other applications, as by changing the antigen recognized or loaded and/or the effector functions, such approaches can be easily transposed to other pathologies. Likewise, banks of personalized B cells could be developed and preserved cryogenically for future needs or in case of disease flares. Moreover, the generation of libraries of B cells with different tumor specificities may increase treatment efficacy by targeting several tumor antigens at the same time.

Overall, advances in B cell modification to fight cancers and diseases in general is coming along very quickly and such approaches might soon come true in the clinics.

## Figures and Tables

**Table 1:** Preclinical mouse studies of B cell transfer with endogenous or compelled tumor antigen presentation for cancer therapy.

Origin of B Cells	Antigen	Number of B Cells (Total)	Route of Infusion	Treatment/Prior Activation	Cancer Model	Results	Ref.
TDLN	Endogenous	1 × 10^6^	iv	IL-2 (infused in vivo)	Metastasis of 4T1 mammary tumor	Anti-4T1- antibodies CXCR4 expression by B cells Reduction of pulmonary metastasis (combined with IL2)	[13]
TDLN	Endogenous	3 × 10^6^	iv	LPS Anti CD40	Metastasis of 4T1 mammary tumor	Anti-4T1- antibodies Generation of T cell responses Reduction of pulmonary metastasis	[14]
TDLN	Endogenous	1 × 10^6^ to 3 × 10^6^	iv	LPS Anti CD40	3-methylcholanthrene-induced fibrosarcoma	Antitumor antigen antibodies Reduction of pulmonary metastasis and tumor size	[15]
Spleens and dCLNs (tumor-bearing mice)	Endogenous	1.5 × 10^6^	iv	CD40 agonist IFNγ BAFF	Glioblastoma	Migration at tumor site and in the SLOs Generation of CD8+ T cell responses 80% of tumor eradication (combined with anti PD-L1, radiotherapy) Memory response	[16]
Spleen	Endogenous (OVA transgenic mice) or peptide loading (pulsed)	1 × 10^5^	iv	CpG Anti-CD40	Thymoma-derived EG-7 cells expressing OVA	Generation of CTL cell responses Protection against tumor growth	[17]
Spleen	Endogenous (frequency of OVA specific cells increased by immunization)	0.1 × 10^6^ to 2 × 10^6^	iv	Feeder cell line expressing CD40L IL4 (+ IL21; CD40L;OVA tetramers to generate plasma cells)	Panc02OVA tumor cells expressing OVA	Migration at tumor site and in the SLOs Anti-OVA antibodies Decreased of tumor growth	[18]
Spleen	Endogenous (frequency of antigen specific cells increase by in vitro culture)	2 × 10^7^	iv	IL-4 IL-21 Feeder cell line expressing CD40L, BAFF, tumor Ag and FasL)	Melanoma metastasis	Anti-HEL antibodies Decreased of tumor growth Increased survival	[19]
Spleen	Tumor peptide loading	5 × 10^6^	iv	CpG Anti-CD40	Thymoma-derived EG-7 cells expressing OVA	Generation of T cell responses Regression of established tumors Increased survival	[20]
Spleen	Tumor peptide loading	1 × 10^6^ to 1 × 10^7^	iv ip sc	Feeder cell line expressing CD40L	Thymoma-derived EG-7 cells expressing OVA Melanoma B16F10 OVA tumor cells	Generation of T cell responses Decreased of tumor growth	[21]
Spleen	Electroporation of RNA encoding tumor Ag	1 × 10^6^	iv sc	LPS	Colorectal cancer	Antitumor antigen antibodies Generation of CD4+ T cell responses Regression of established tumors Increased survival	[22]
Spleen	Viral delivered (Adenovirus)	2 × 10^6^	iv	CFm40L harbored on adenovirus	E6/E7-expressing TC-1 cell line human Her-2/neu-expressing CT26 cell line murine Her-2/ neu-expressing CT26 cell line	Generation of CTL cell responses Decreased of tumor growth Increased survival	[23]
Spleen	Viral delivered (adenovirus)	2 × 10^6^	iv	α-galactosylceramide	Her2/neu-expressing transfectoma cell line CT26-hHer2 Her2/neu-expressing SK-Br-3 human breast carcinoma	Migration in the SLOs Antitumor antigen antibodies Generation of CD8+ T cell responses	[24]
Spleen	Viral delivery (adenovirus)	2 × 10^6^	iv	α-galactosylceramide	Her2/neu-expressing transfectoma cell line CT26-hHer2 or CT26-hp95Her2	Antitumor antigen antibodies Generation of CTL cell responses Decreased of tumor growth Increased survival	[25]

TDLN: tumor-draining lymph node. LPS: lipopolysaccharide. CD: cluster of differentiation. IL: interleukine. IFNg: interferon gamma. BAFF: B cell activating factor. OVA: ovalbumin. iv: intravenous. ip: intraperitoneal. sc: subcutaneous.

## Data Availability

Not applicable.

## Appendix A. Transgenic Mouse Strains with BCR Specific to a Given Antigen

As it may be complicated to isolate high amounts of tumor antigen-specific B cells, many studies take advantage of the BCR-specific mouse strains that have been developed for proof-of-concept studies [78]:

- SWHEL transgenic mice: hen egg-white lysozyme antigen [79]
- QM transgenic mice: 4-hyroxy-3-nitrophenyl acetyl (NP) antigen [80]
- OBI-transgenic mice: ovalbumin antigen [81]

## Appendix B. Advantages of Immune Cells for Synthetic Biology

Traffic to inflamed areas

Natural sensing capacities

Proliferation in response to specific cues

Generation of memory cells (long-term)

Communication with the immune system
